# Socioeconomic inequalities linked to the transitioning to neurocognitive disorders and mortality

**DOI:** 10.1038/s41598-024-74125-w

**Published:** 2024-11-01

**Authors:** Aswathikutty Gireesh, Amanda Sacker, Anne McMunn, Rikesh Bhatt, Dorina Cadar

**Affiliations:** 1 Department of Epidemiology and Public Health, University College London, 1-19 Torrington Place, London, WC1E 7HB UK; 2 Department of Behavioural Science and Health, Institute of Epidemiology and Public Health, University College London, 1-19 Torrington Place, London, WC1E 7HB UK; 3 Institute of Health Informatics, University College London, 222 Euston Road, London, NW1 2DA UK; 4 Department of Neuroscience, Centre for Dementia Studies, Brighton and Sussex Medical School, Trafford Centre, Brighton, BN1 9RY UK; 5 Department of Primary Care and Public Health, Brighton and Sussex Medical School, Brighton, BN1 9PX UK; 6 Evaluation and Epidemiological Science, UK Health Security Agency, London, UK

**Keywords:** Neuroscience, Medical research, Risk factors

## Abstract

Research on socioeconomic position (SEP) and mild neurocognitive impairment, considered a transient state between normal cognitive function and dementia is limited. The purpose of this study was to determine the role of SEP in transitioning between different cognitive states and mortality risk. Using nationally representative English data and utilising a multistate model association between SEP and the risk of transitioning from no cognitive impairment (NOCI) to Cognitive impairment no dementia (CIND), dementia and death were investigated. The potential reverse transition from CIND to NOCI was also explored. The probabilities of transitioning between cognitive states and time spent in each state differed significantly between those with lower and higher levels of SEP. Higher wealth was associated with a reverse transition from CIND to NOCI [HR = 1.56, CI (1.42,1.72)]. Socioeconomic advantage might protect against the progression to the early stages of neurocognitive disorders (CIND) and facilitate the potential reversion from mild cognitive impairment to a healthy cognitive state in later life. Lower levels of education affect the risk of mortality after the onset of dementia.

## Introduction

There has been a consistent upward trend in the overall mortality rate attributed to dementia and Alzheimer’s disease in the UK since 2001, with dementia and Alzheimer’s disease-related deaths accounting for 12.5% of the total deaths in England and Wales, according to the latest census^1^. Furthermore, dementia is projected to double within the next twenty years^2^. Thus, there is an imperative need to prioritise prevention and early detection among older adults at risk. Socioeconomic position, often measured by education, occupation, wealth, and income, has been recognised as a determinant factor in relation to dementia risk^3^. Three recent meta-analyses^4–6^ observed a strong association between lower levels of education and dementia risk among all socioeconomic indicators studied. While two of these meta-analyses initially found an association between lower occupational positions and lower income levels with dementia risk, upon adjustment for confounders, these associations attenuated^4,6^. The relationship between socioeconomic inequalities and time spent with cognitive impairment is another area of research highlighted in literature and some evidence suggests that individuals from lower socioeconomic backgrounds spent a larger proportion of their later years with cognitive impairment compared to their higher socioeconomic counterparts^7–9^, although contradictory findings exist^10–12^. Understanding the impact of wealth on neurocognitive disorders presents a challenge, given the limited number of studies examining it, yet the available studies indicate a positive link between higher wealth and lower dementia risk^13–15^. However, the role of socioeconomic inequalities in transitioning from a healthy cognitive state to mild neurocognitive states such as mild cognitive impairment (MCI) and dementia over time is poorly understood.

The process of cognitive ageing follows a continuum from a normal cognitive state to dementia with an intermediate phase of MCI^16^. In contrast to dementia, where other cognitive skills and the ability to live independently are affected, MCI is characterised by cognitive impairment without dementia or functional impairment^17^. Clinical evidence indicates that individuals with MCI experience similar characteristics and types of neuropathological changes as seen in mild dementia^18^. Studies also suggest that around 12% to 15% of individuals with MCI could develop dementia each year^19^. Population-based studies are thus critical when estimating which older individuals are at an increased risk of MCI and dementia. This can help elucidate the rates of progression over time and provide vital information for public policy.

Except for two^20,21^, previous UK studies have primarily focused on either MCI or dementia outcomes, which limits our understanding of the role of socioeconomic risk factors in transitioning between various cognitive states. One study using the data from Whitehall II showed a lower risk of transitioning to mild neurocognitive states associated with higher education^20^. Consistent findings were seen in a second study using data from the Cognitive Function and Ageing Study (CFAS)^21^. Results indicated that higher cognitive reserve (characterised by higher levels of education and occupation) could offer a buffer against forward transitions to mild neurocognitive disorder and facilitate reverse changes to a healthy cognitive state^21^. A few other studies^22–26^ also provide supportive evidence for the role of education in the transition between healthy cognitive and mild neurocognitive states using a multistate framework. In addition, a meta-analysis^27^, including 17 studies, found that individuals with higher education are more likely to return to a healthy cognitive state from MCI; however, results were inconclusive due to the sparsity of the reviewed studies and heterogeneity in the samples included. Nevertheless, studies exploring the multistate transitional nature of neurocognitive disorders have often disregarded more reliable markers of socioeconomic position in later life, such as wealth. In general, studies in this line of research have been limited to the analysis of non-representative^25,26,28^, small^26,28^, occupational^20^ or clinical samples^27^, making their generalisability difficult. More importantly, the earlier UK-based studies^20,21^ seldom used standardised population-based neurocognitive measures and instead relied on cognitive screening tools.

We aim to determine whether there is an association between socioeconomic markers and transitioning between different cognitive states and ultimately to death using a large nationally representative study of middle-aged and older English adults. To effectively capture the socioeconomic disadvantage in later life, wealth indicator will be considered alongside education and occupation. This work also aims to estimate the time spent in each cognitive state in relation to each socioeconomic marker (education, occupation, and wealth).

## Methods

### Data

The English Longitudinal Study of Ageing (ELSA) is an ongoing longitudinal survey of the ageing population in England. ELSA is a representative sample of men and women 50 years and older living in England, which started in 2002–2003, with refreshment samples at different waves. For these analyses, we considered the longitudinal data available from wave four (2008–2009) to wave nine (2018–2019), with data on outcomes collected at six different time points. Wave 4 was chosen as the baseline as it had the maximum sample size (due to a large refreshment sample at this wave). Among the core sample of 9821 participants at baseline, 8442 eligible participants with 76,242 data points were selected. A flowchart depicting sample selection is provided in the supplementary eFigure S2. The datasets generated and/or analysed during the current study are publicly available via the UK Data Service (https://www.ukdataservice.ac.uk) except for the mortality data. Ethical approval for each of the ELSA waves was granted from the National Research Ethics Service (London Multicentre Research Ethics Committee) (MREC/01/2/91) (http://www.nres.npsa.nhs.uk). All participants gave informed consent at each of the recruitment waves to participate in the study, and all methods were conducted in accordance with the relevant guidelines and regulations. We used the STROBE cohort checklist when writing our report.

### Study variables

#### Neurocognitive disorders

The presence of neurocognitive disorders was operationalised in ELSA using the consensus criteria according to the Diagnostic and Statistical Manual of Mental Disorders (DSM-5), Ref.^29^ following a diagnostic algorithm implemented in CFAS, Ref.^30^ which has been shown to have good predictive accuracy for dementia in population-based settings. Separate cognitive status groups were derived based on the self-reports of a physician’s dementia diagnosis, objective tests for cognitive impairment, subjective reports of memory complaints, and functional impairment. The diagnostic groups derived were (i) No Cognitive Impairment (NOCI), (ii) Mild Cognitive Impairment (MCI), (iii) Other Cognitive Impairment no dementia (OCIND), and (iv) Dementia. Since MCI was only a small group in this study, OCIND was regrouped with MCI to create a Cognitive Impairment No Dementia (CIND) group. Therefore, for the main analysis, we used the following categories: (1) NOCI, (2) CIND, and (3) Dementia. For more details on the classification criteria for neurocognitive disorders, see the previously published paper^31^ and the Supplementary file.

#### Socioeconomic indicators

Socioeconomic position was measured using three indicators: education, occupation, and non-pension household wealth. These were based on self-reports in computer-assisted personal interviews collected at wave four. The highest attained educational qualification was classified as: “low (primary education or less), middle (secondary education), and high (tertiary education)”. The occupational class included the following categories: “managerial and professional occupations, intermediate occupations, and semi-routine/routine/manual occupations”^31,32^. Total non-pension household wealth was categorised into wealth tertiles, with tertile 1 being the most deprived and tertile 3 being the most affluent.

### Covariates

Potentially significant covariates were demographic factors (age, sex, and marital status). Information on covariates was collected at wave 4. Sex was grouped as: “male” and “female” and marital status was grouped as “partnered” (married or partnered) and “not living with a partner” (single, legally separated, divorced, widowed). Being “male” and “married” were used as the reference groups.

#### Statistical analyses

**Fig. 1:**
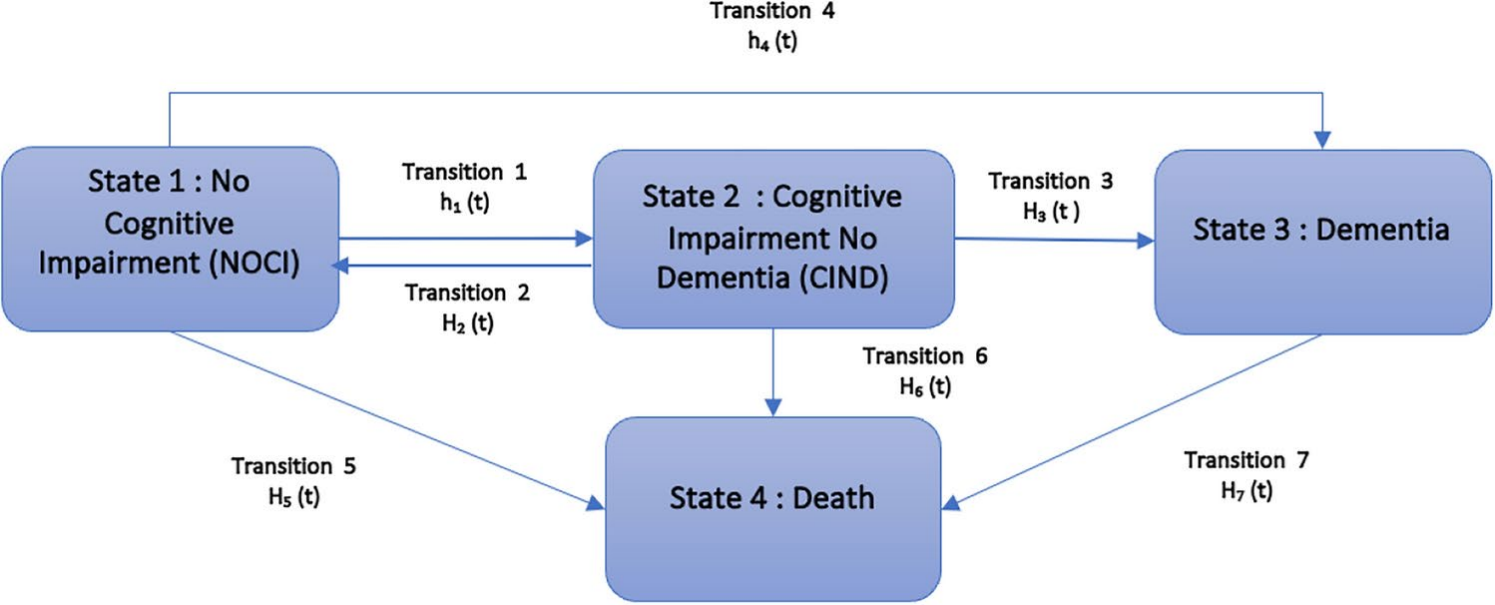
The conceptual model of the statistical analyses employed with three cognitive states and seven transition probabilities considered between different cognitive states with death as an absorbing state. The states are represented with boxes and numbered 1, 2, 3, and 4. Each transition is represented with an arrow, indicating the probability of transitioning from one state to another.

First, baseline socioeconomic factors and covariates are summarised by NOCI and CIND using descriptive statistics. A continuous time Markov model was employed to model the transition probabilities between different cognitive states: NOCI, CIND, and dementia, over a 10-year period using wave 4 as the baseline. The Markov model used a piecewise-constant approximation for age dependency^33^. Further details are provided in the Supplementary material. If a state can continue to transition to another state, it is classified as a transient state; otherwise, it is considered an absorbing state. There are seven possible transitions among the four states, and death was considered an absorbing state, as illustrated in Fig. 1.

We plotted the observed prevalence versus the predicted prevalence from the fitted model over time to check the model goodness-of-fit (Supplementary eFigure S3). Transition-specific hazard ratios and 95% confidence intervals (CI) are estimated to determine how each socioeconomic indicator is associated with transition risk across different cognitive states. In addition, we calculated the probabilities of entering each state and the total mean time spent in each state separately for education, occupation, and wealth at ages 60 and 80. Finally, we estimated the average length of stay in various states and the sojourn time. All models are adjusted for age, sex, and marital status except for the transition from NOCI to dementia and CIND to dementia. In all analyses, socioeconomically disadvantaged groups were used as a reference group for comparison against other levels. Due to convergence issues, the inclusion of more covariates was challenging. Differences between study members who had complete neurocognitive data and those who did not were analysed by covariates (Supplementary Table S4). Standard errors are estimated from the Hessian matrix evaluated at the optimum (Supplementary Table S5). The ‘msm’ package of R version 4.2.0 was used to build the multistate Markov model^34^.

#### Role of the funding source

The funders had no role in study development, data analyses, interpretation, or writing of the report.

## Results

### Sample characteristics

Participants were followed up for a mean of five years. The baseline sample at wave 4 was predominantly female (55.74%) and married (65.30%). Detailed descriptive statistics can be found in Table 1.

**Table 1:** Baseline demographic and socioeconomic characteristics of the analytical sample (n = 8442).

Study variables	Mean (SD)/n (%)
Age	67 (9.5)
Sex	
Male	3779 (44.76)
Female	4663 (55.74)
Marital status	
Single/divorced	2929 (34.70)
Married	5513 (65.30)
Education	
Low	2348 (27.81)
Middle	4568 (54.11)
High	1526 (18.07)
Occupation	
Routine/Manual	2619 (31.02)
Intermediate	2975 (35·24)
Managerial/Professional	2848 (33.73)
Wealth	
Lowest tertile	2769 (32.80)
Middle tertile	2849 (33.74)
Highest tertile	2824 (33.45)

### Transition frequency and transition probabilities

**Fig. 2:**
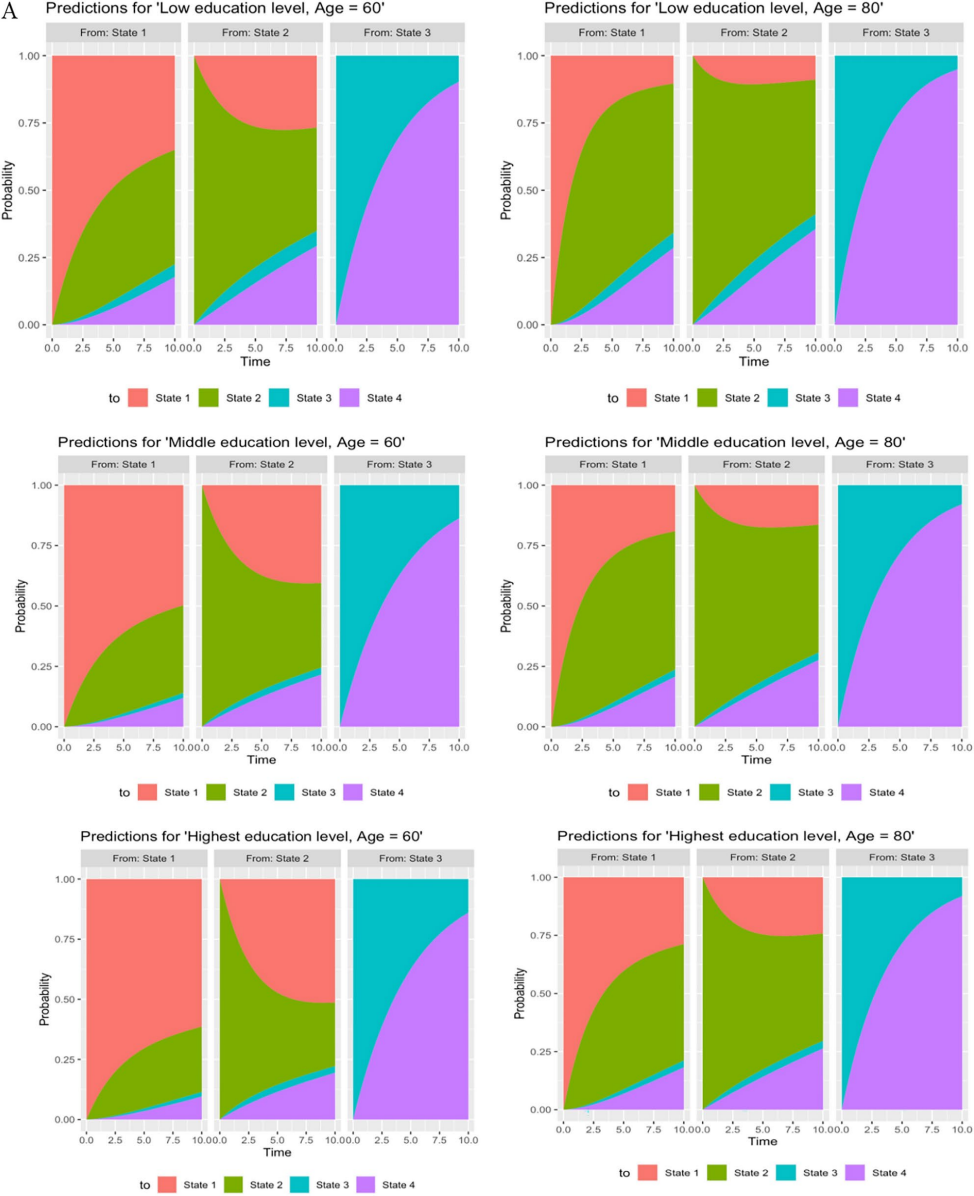

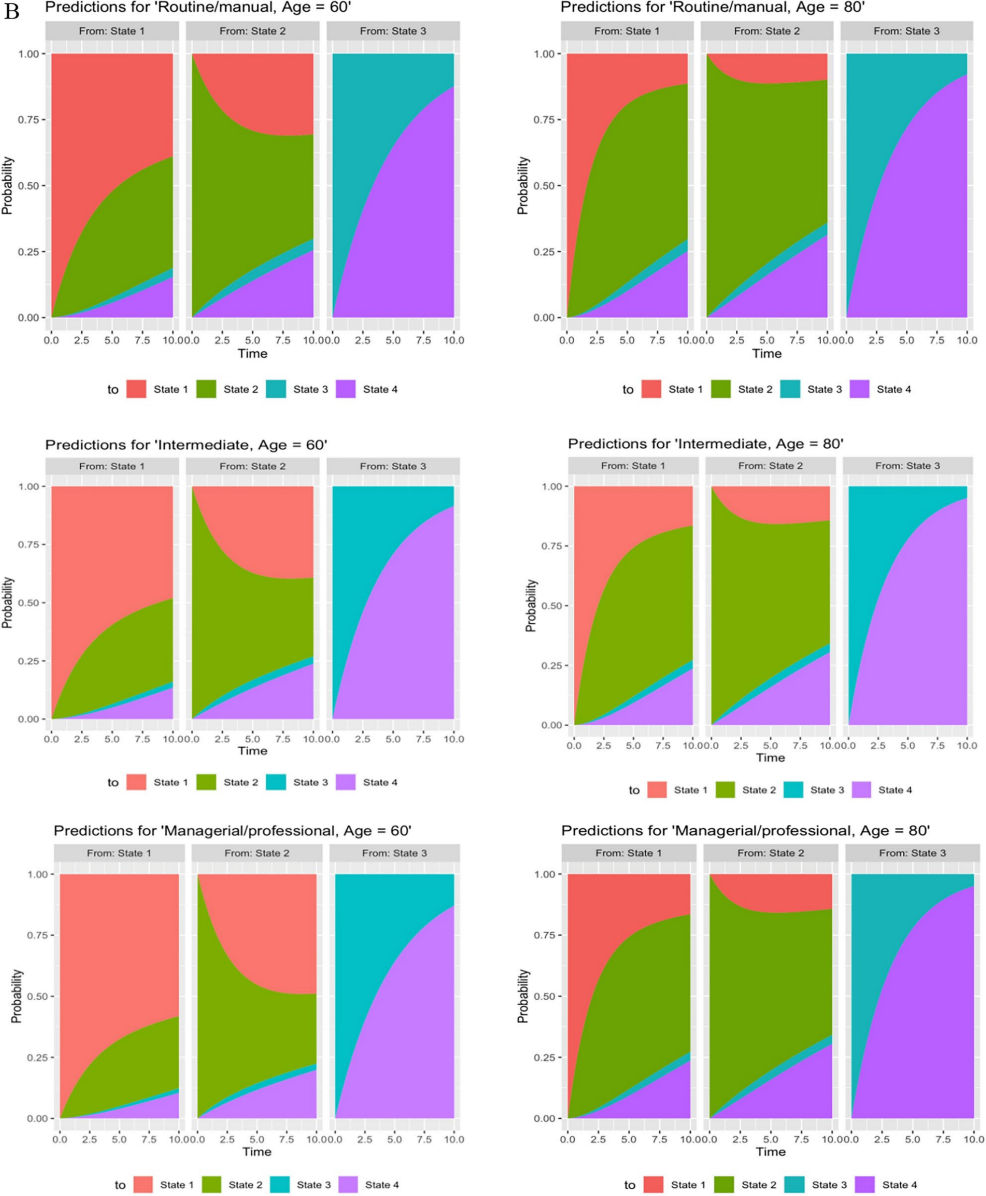

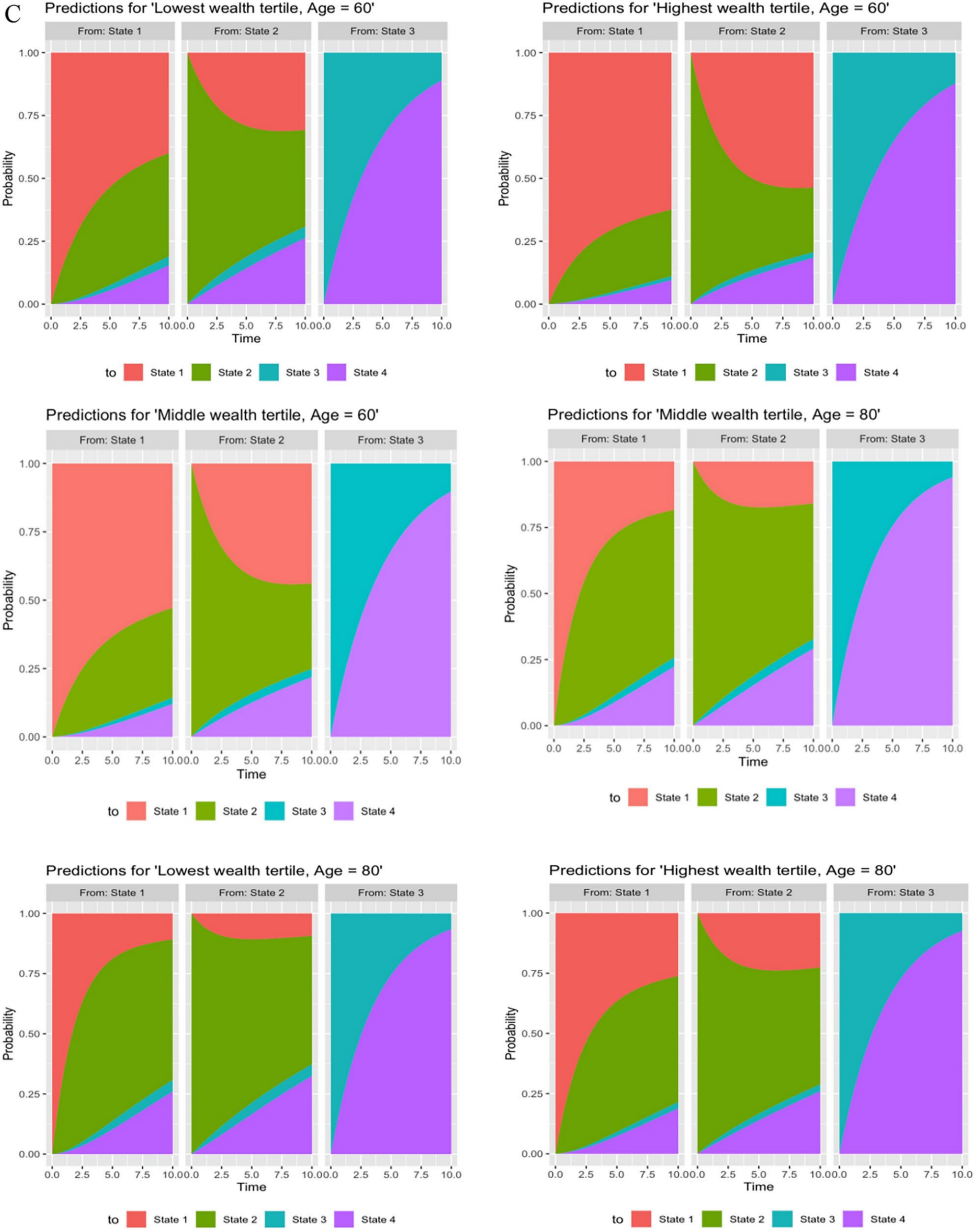
(**A**) Stacked predicted transition probabilities for different education groups. (State 1: No Cognitive Impairment, State 2 = Cognitive Impairment no dementia, State 3 = Dementia, State 4 = Death). (**B**) Stacked predicted transition probabilities for different occupation groups. (State 1: No Cognitive Impairment, State 2 = Cognitive Impairment no dementia, State 3 = Dementia, State 4 = Death). (**C**) Stacked predicted transition probabilities for different wealth groups. (State 1: No Cognitive Impairment, State 2 = Cognitive Impairment no dementia, State 3 = Dementia, State 4 = Death).

During the study period, 3898 transitions occurred from NOCI to CIND state and 3361 transitions from CIND to NOCI state. The least number of transitions occurred between NOCI and dementia (n = 25) and transitions from CIND to dementia were higher (n = 336). A range of transitions to the terminal state of death were observed. Specifically, there were 1231 cases with transitions from CIND to death, reflecting a higher number of occurrences. Additionally, 295 cases transitioned from NOCI to death, representing a moderate number of transitions. However, only 192 cases progressed from dementia to death, indicating a lower number of transitions. There were seven possible pathways for participants during the ten-year follow-up time with a transition probability matrix for the unadjusted model. Nearly 17% transitioned from NOCI to CIND, and 11% underwent a reverse transition to NOCI from CIND (transition probability = 0.11, CI 0.10, 012). The probability of transitioning from NOCI to death and dementia was found to be remarkably low. On the other hand, dementia to death (transition probability = 0.21, CI 0.18,0.24) was found to be more prevalent. Given the fitted multistate model, adjusted transition probabilities were also predicted for each covariate pattern at ages 60 and 80. The stacked probabilities of state occupancy are displayed in Fig. 2A–C. The corresponding transition probabilities for these figures are provided in Supplementary Tables S1–S3. When transitions were tracked over ten years or by age, differences in transition probabilities among participants from the most and least advantaged socioeconomic positions diverged further.

### Transition probabilities in relation to education

After ten years, the highest education level was associated with lower probabilities of transitions to CIND [Age 60, lowest: 0.42 (0.40, 0.44) vs highest: 0.27 (0.25, 0.29)] and dementia from NOCI compared to those with the lowest education level. Similarly, the probabilities of transitioning from CIND to dementia also differed between those with primary education or less and those with a degree education or more. The reverse transition from CIND to NOCI was more likely in participants with higher education than those with lower levels of education. Moreover, higher education level was inversely associated with the transition probability from dementia to death, with individuals having lower education levels exhibiting a higher risk of mortality.

### Transition probabilities in relation to occupation

Participants who had a managerial/professional level occupation had the lowest probability of CIND [Age 60, Routine/manual occupation: 0.43 (0.40,0.44) vs Managerial/professional: 0.36 (0.34,0.37)] and dementia by ten years. Participants in a more disadvantaged occupational class had a higher probability of moving to dementia from NOCI and CIND. More advantaged occupational positions significantly increased the probability of reversing back to NOCI compared with the manual/routine occupational class. Additionally, routine/manual occupations exhibited higher probabilities of transitions to dementia or death compared to professional-level occupations.

### Transition probabilities in relation to wealth

Participants in the bottom wealth tertile had a lower probability [Age 60, 0.41 (0.39,0.43)] of transitioning from NOCI to CIND in ten years, compared with lowered probability [Age 60, 0.26 (0.24,0.28)] among participants in the top wealth tertile. The probability of reverse transitions was stronger for the wealth indicator [top wealth tertile—Age 60, lowest tertile: 0.30 (0.28,0.32) vs highest tertile: 0.53 (0.51,0.56)]. The differences in transition probabilities between different tertiles of wealth for transitions from NOCI to dementia, dementia to death, and CIND to dementia were small. Increasing age was strongly associated with an increased likelihood of transitions, except for CIND to NOCI.

### Risk of transition between cognitive states

Adjusted hazard ratios and 95% confidence intervals of each transition are shown in Table 2. Education was a strong factor in most of the transitions, and the highest level of education was associated with a 43% reduced risk of transitioning from NOCI to CIND, a 69% reduced risk of transitioning from CIND to dementia, and a 39% reduced risk of transitioning from dementia to death compared to those with the lowest level of education. Similarly, higher education was associated with an increased risk of backward transition from CIND to NOCI (HR = 1.81, 95% CI 1.61, 2.04). A more advantaged occupational position was also associated with a lower risk of transitioning from NOCI to CIND. Compared to manual workers in state CIND, professional workers had an 81% higher chance of reverting to NOCI. Additionally, individuals in higher education levels, managerial/professional occupations, and highest wealth tertiles were at lower risk of transition from CIND to dementia compared to their counterparts in lower socioeconomic positions. Higher wealth was also associated with reversion from CIND to NOCI. The association between neither occupation nor wealth with the transitions from dementia to death was statistically significant. No socioeconomical indicator was associates with transition from NOCI to dementia. However, there was reduced power to observe the association of SEP on the transition progression probability from CIND to death and NOCI to death.

**Table 2:** Association of baseline socioeconomic factors with the risk of transitions in ELSA during the 10 years follow-up.

Variable	NOCI to CIND	CIND to NOCI	CIND to Dementia	NOCI to Dementia	Dementia to Death
	HR (95% CI)	HR (95% CI)	HR (95% CI)	HR (95% CI)	HR (95% CI)
Education					
Low					
Middle	0.73 (0.67, 0.80)	1.37 (1.24, 1.51)	0.38 (0.31, 0.47)	0.81 (0.11, 5.94)	0.69 (0.55, 0.87)
High	0.57 (0.52, 0.64)	1.81 (1.61, 2.04)	0.31 (0.22, 0.45)	0.91 (0.10, 8.49)	0.61 (0.39, 0.93)
Occupation					
Routine/Manual					
Intermediate	0.82 (0.75, 0.89)	1.46 (1.32, 1.62)	0.80 (0.63, 1.02)	0.91 (0.15, 5.44)	1.03 (0.80, 1.34)
Managerial/Professional	0.67 (0.61, 0.73)	1.81 (1.64, 2.00)	0.63 (0.48, 0.82)	0.88 (0.16, 4.80)	0.96 (0.71, 1.29)
Wealth					
Lowest tertile					
Middle tertile	0.86 (0.79, 0.93)	1.29 (1.17, 1.43)	0.98 (0.77, 1.24)	0.90 (0.15, 5.44)	1.18 (0.92, 1.53)
Highest tertile	0.68 (0.63, 0.74)	1.56 (1.42, 1.72)	0.74 (0.56, 0.97)	0.87 (0.16, 4.89)	0.98 (0.74, 1.31)

*HR* Hazard Ratio, *CI* Confidence Intervals, *NOCI* No Cognitive Impairment, *CIND* Cognitive Impairment no dementia.

### The expected total length of time spent in each state

**Fig. 3:**
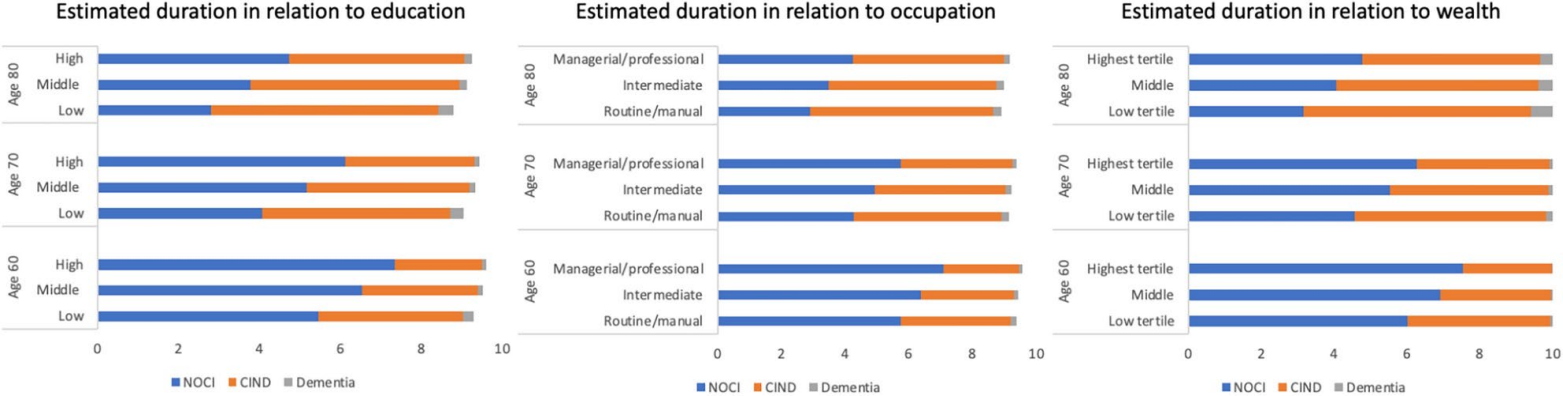
Estimated duration of each cognitive state in years, separately for education, occupation, and wealth categories at ages 60, 70, and 80. *NOCI* No Cognitive Impairment, *CIND* Cognitive Impairment no dementia.

Average years spent in each state during 10 years of follow-up are presented for the three socioeconomic indicators in Fig. 3. Compared with participants who had a lower level of education, degree-level educated participants had a longer expected mean time spent in the NOCI state at all ages (7.35 at age 60 v/s 4.73 at age 80). Compared with participants who were less socioeconomically advantaged, participants who were well-educated, professionally qualified, and wealthy at age 80 years had a shorter expected remaining time spent in the CIND state and dementia state. Notably, the expected total time spent in the NOCI state increased in a stepwise pattern, and the expected total time spent in CIND and dementia states decreased in a stepwise pattern with an increase in educational, occupational, and wealth positions. Sojourn times also varied by socioeconomic indicators, with individuals in socioeconomically advantaged positions experiencing longer durations in NOCI and lesser durations in CIND and dementia compared to those in lower socioeconomic positions at age 80 (see supplementary Table S6).

## Discussion

In a longitudinal cohort of English adults aged 50 and over, the association between socioeconomic risk factors and the risk of progressing to neurocognitive disorders in later life was studied over a ten-year period. A significant portion of older adults was likely to undergo forward and backward transitions between healthy cognitive states and neurocognitive disorders such as mild cognitive impairment. Results showed that highly educated professionals, higher occupational groups, and wealthy participants had lower hazards of transitioning from NOCI to CIND and from CIND to dementia than participants with lower levels of education, occupation, or wealth. Individuals from socioeconomically advantaged groups demonstrated a significantly higher chance of reverting from CIND to NOCI. It is noteworthy that while education showed significant and consistent associations across most transitions except NOCI to dementia, occupation and wealth did not exhibit associations for transitions from NOCI to dementia and dementia to death. On average, socioeconomically advantaged groups spent more time in cognitively healthy states and less time in mild to severe cognitive impairment states than socioeconomically disadvantaged groups.

The results of this study provide some interesting insights. The transition rates between NOCI and CIND states fall within the wide range reported in a prior meta-analysis^19^. However, progression rates from a healthy cognitive state to dementia were lower, and this conflicts with findings of earlier studies, which have shown that the rate of transition over a 10-year period could be 8–15%^35,36^. Based on HuiPing Xue’s meta-analysis findings, the reversion rates from MCI to a healthy cognitive state averaged 28%, which is higher than that observed in this study. Nevertheless, comparing our results with other studies is difficult because of the different methodologies, age ranges, and populations studied. It is also possible that, being a generally healthy cohort, a significant proportion of this study population might have dropped out before observing any cognitive impairment or dementia.

The observed association between education and the risk of progressing to CIND is in agreement with the results of earlier studies using a similar analytical strategy^21,26,28^. Education also predicted the progression to dementia from CIND and was found to be associated with more years spent without cognitive impairment and fewer years with CIND and dementia in this study. Our study contrasts with the previous finding, which reported no association between education and the time spent in cognitive impairment^11^. Moreover, unlike another earlier UK study^10,12^, our research indicates a significant association, showing that individuals with higher education levels live less years with cognitive impairment. These discrepancies may arise from differences in study methodologies, or changes in educational contexts. However, further research is required to explore and clarify the mechanisms behind these associations.

The findings provide support for the notion that complex managerial or professional-level jobs can be associated with a lower risk of progression to CIND from NOCI and CIND to dementia. A prior study employing multistate modelling found a similar association with the risk of moving to a mildly impaired state; however, the occupational level did not contribute to the progression toward dementia in that study^21^. Another multicohort study found similar associations between a higher occupation level and a lowered risk of transitioning from a normal cognitive state to mild cognitive impairment^20^. However, the association between occupation and transitioning from mild impairment to severe impairment was significant only in the Longitudinal Aging Study Amsterdam (LASA)^20^.

We identified a higher transition rate for reversal of CIND to NOCI among all three socioeconomically advantaged groups. Education emerged as a significant predictor of CIND reversion, which is in line with previous findings^24,27^; however, the contribution of occupation and wealth to the relative process of transitioning from a healthy cognitive state to neurocognitive disorders represents new knowledge and contributions.

In fact, to the best of the authors’ knowledge, this is the first study to test the association of wealth with neurocognitive transitions to MCI, dementia, and mortality. Wealth seems to be strongly associated with transitions from NOCI to MCI and from MCI to dementia, even after adjustment for confounders. The process through which socioeconomic factors protect against early progression to MCI is not fully elucidated; however, different indicators may affect health through different mechanisms^37^. One probable explanation for the observed results could come from Stern’s cognitive reserve hypothesis, which postulates that highly educated older adults with no cognitive impairment might have a high cognitive and brain reserve that is able to mask and overcome the pathological burden associated with dementia^38^. It is also possible that increased wealth and better occupation increase access to resources, contributing to higher cognitive reserve^39^. Another possibility is that this may simply reflect the underlying socioeconomic gradient in physical health^40^ and reflect better access to health service providers and early treatments for various chronic conditions (e.g., hypertension, diabetes, cardiovascular disease) contributing to dementia risk. Occupation can also influence health through exposure to physical hazards and access to work benefits such as insurance and pensions^41,42^. Finally, another mechanism could be operating via a stress pathway, i.e., socially disadvantaged groups can suffer chronic stress, stimulating glucocorticoid release, thus affecting the hippocampus area associated with the memory centre^43^.

### Strength and limitations

The work presented has a number of strengths and limitations.

In terms of limitations, we must first acknowledge that dementia diagnosis, although made by a physician, was self-reported. Second, participants were followed for ten years only, which might not be long enough to detect the process of transitioning from normal cognitive status to mild cognitive impairment, emerging dementia, and mortality cases. Third, due to power issues, we were unable to consider all potential confounders that could have influenced the transitions between cognitive states. Fourth, this analysis may be limited by the absence of certain transitions or states due to missing data. There could be also potential miss-classification of cognitive status. Fifth, the analysis did not consider all the subtypes of neurocognitive disorders and because of low case numbers in certain cognitive states. Sixth, the study may be subject to selection bias, as individuals with worse cognitive health are more likely to be lost to follow-up. Finally, ethnicity was not adjusted for in the analysis, given the predominantly white composition of the ELSA sample.

Despite these limitations, the study has made substantial contributions. The study population used in the current analyses is a representative sample of the English population aged 50 and older living in private households. This represents a unique and rich resource of information on the dynamics of health and economic circumstances in the English population that offered an excellent opportunity to investigate the role of socioeconomic differential in relation to transitioning to neurocognitive disorders and mortality. Second, not many studies have considered multistate modelling and the transitional nature of neurocognitive impairment and death, nor have they used a reliable marker of later-life SEP in a population-representative epidemiological study. The consideration of the multifaceted nature of SEP has provided a more nuanced understanding of the various dimensions of SEP and how they impact cognitive health^37,44^. We have also considered both subjective and objective measures of cognition to ascertain neurocognitive disorders. Furthermore, Markov modelling is a complex tool for analysing population-level cognitive change in transitioning to impairment. Through the multistate model, the average time in each state and the transition probabilities from one state to another were estimated, thereby providing valuable information for targeted preventive interventions in the progression of neurodegeneration in older people.

### Conclusion

Results from this study provide a new and valuable understanding of the role of socioeconomic markers in relation to transitioning to neurocognitive disorders and mortality. The current associations between SEP indicators and transitioning to neurocognitive disorders and mortality may provide new insights into the importance of socioeconomic inequalities in transitioning to mild neurocognitive disorders by showing persistent inequalities by education, occupation, and particularly wealth.

## Supplementary Information

Supplementary Information.

## Data Availability

The datasets generated and/or analysed during the current study are publicly available via the UK Data Service (https://www.ukdataservice.ac.uk) except for the mortality data.
