# Supplementary material for: Socioeconomic inequalities linked to the transitioning to neurocognitive disorders and mortality
Source: Sci Rep. 2024 Nov 1;14:24690. doi: 10.1038/s41598-024-74125-w (PMC11530460; doi:10.1038/s41598-024-74125-w)
Supplement: Supplementary file 1 — Supplementary Information. [file 41598_2024_74125_MOESM1_ESM.docx]

**SUPPLEMENTARY MATERIALS**

**Multistate continuous time Markov model with piecewise-constant approximation**

In this study, continuous Markov time model was fitted using the piecewise-constant approximation method for hazard models.

The intensity matrix, *Q(t)*, which describes the multistate model, is given by

$$Q\left( t \right) = \left( \begin{matrix} {-h}_{1}\left( t \right)-h_{2}\left( t \right)-h_{3}(t) & h_{12}(t) & h_{13}(t) & h_{14}(t) \\ h_{21}(t) & {-h}_{1}\left( t \right)-h_{2}\left( t \right)-h_{3}(t) & h_{23}(t) & h_{24}(t) \\ h_{31}(t) & h_{32}(t) & {-h}_{1}\left( t \right)-h_{2}\left( t \right)-h_{3}(t) & h_{34}(t) \\ 0 & 0 & 0 & {-h}_{4}(t) \end{matrix} \right)$$

,

where $h_{n}(t)$ are the hazard functions in Figure 2. The hazard functions, $h_{n}(t),$ are Gompertz hazard functions

$$h_{n}(t) = \exp(\lambda_{n}+\beta t+\boldsymbol{\alpha}_{\boldsymbol{n}} \mathbf{Z})$$

t is age, and the variable **Z** is the time-constant covariate values. $\lambda_{n}$, $\beta$ and $\boldsymbol{\alpha}_{\boldsymbol{n}}$ are parameters of the model.

The general assumption is that the probability distribution of the cognitive status at the future wave depends only on the present wave or possibly the risk factors and is thus assumed to have the Markov property. ^1^ It is important to note that the fitted model is not piecewise constant because piecewise-constant models assumes that the hazard is constant from one interval to the next ^2^. The method employed in this study is piecewise-constant approximation which assumes that estimate is constant within individually observed time intervals (which is a year in this study) but estimates are allowed to vary between intervals according to the parametric Gompertz distribution^3^. The main advantage of the piecewise-constant hazard approximation is that it accounts for the age-dependency of the Markov process ^4^. Another advantage is the ease of parameter estimation without the need to specify interval-specific hazard parameters ^2^.

**Diagnostic algorithm for the derivation of neurocognitive disorders**

The classification of neurocognitive disorders was derived from the following measures of cognition available in the ELSA dataset:

1. Objective cognitive impairment

We selected cognitive tests available at all waves (memory, orientation, and verbal fluency). Memory was assessed using a word recall test, where a recording of ten familiar words was first played to the participants. Participants were asked to remember these words and recall them at once (immediate word recall) and after approximately five minutes (delayed word recall). A summed total memory score was created with scores ranging from 0-20, with higher scores indicating greater memory. Orientation in time was assessed through standard questions on the day, month, year, and day of the week. The answers were combined to derive a summed “total orientation score” with scores ranging from 0-4, with higher scores indicating greater orientation. Verbal fluency test was conducted with an animal naming task where participants were given one minute to name as many animals as possible. The number of animals named was recorded. Scores ranged from 0 to 50, with higher scores indicating a higher number of animals named.

A memory composite score was calculated by averaging across z-scores (based on the mean and standard deviation) for the immediate and delayed recall tests. A non-memory composite score was calculated by averaging across z-scores (based on the mean and standard deviation) for orientation and verbal fluency. Objective cognitive impairment for each cognitive domain 1 SD (Standard Deviation) below the mean on the age- and sex-adjusted cognitive z-score for that domain at each wave. A classification of MCI required impairment in any of the two domains used (31). MCI was further classified as impairment in memory performance only (amnestic MCI), non-memory impairment only (non-amnestic MCI) and multi-domain MCI with more than one domain affected (multiple amnestic MCI).

1. Subjective memory complaint

Subjective memory was assessed by asking participants to rate their current memory ability on a 5-Likert scale, where 1 = poor memory, 2 = fair memory, 3 = good memory, 4 = very good memory, and 5 = excellent memory. Those rating their memory as poor, or fair were identified as having a subjective memory complaint.

1. Functional impairment (FI)

Functional impairment was assessed using 6 Activities of Daily Living (ADLs) items; (a) dressing (b) eating (c) using the toilet (d) bathing and showering (e) getting in and out of bed (f) walking across a room. Functional impairment was defined as needing help with one or more ADLs.

1. Dementia

Dementia was defined from a combination of self or proxy-report physician diagnosis of dementia or Alzheimer’s disease, or a score above the threshold of 3.38 on the 16-question Informant Questionnaire on Cognitive Decline in the Elderly, known to have high specificity and sensitivity (Jorm 1994, Quinn et al. 2014).


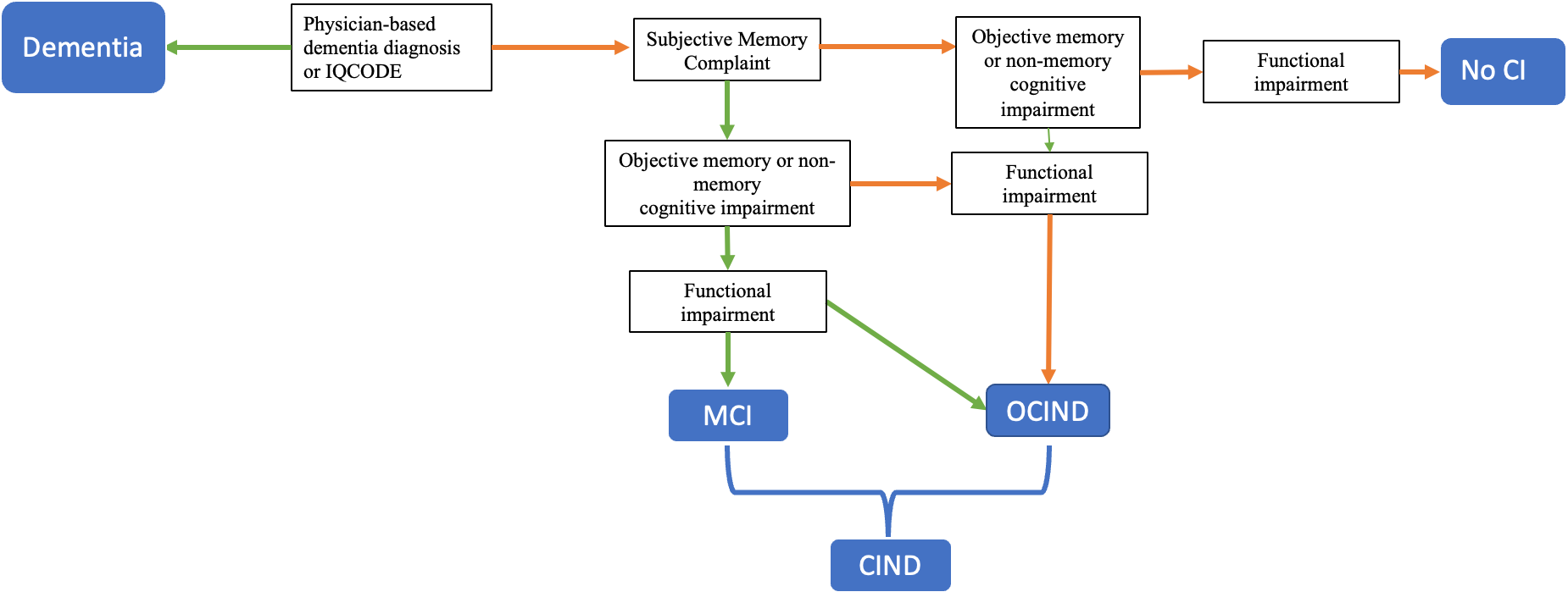


*eFigure S1: Flow chart describing the classification of neurocognitive disorders*

*NOCI= No Cognitive Impairment, MCI= Mild Cognitive Impairment, OCIND= Other Cognitive Impairment, no dementia, CIND= Cognitive Impairment, no dementia,*

**Classification of neurocognitive disorders**

To be classified as NOCI, individuals had to fulfil the following criteria: (i) no dementia, (ii) normal general cognitive functioning, (iii) no functional impairment and (iv) normal memory and non-memory test performance (v) no self-reported memory complaint (Stephan et al. 2011). For classification of MCI, individuals had to fulfil the following criteria: (i) no dementia, (ii) subjective report of memory loss, (iii) no functional impairment and (v) objective memory or non-memory impairment (defined using percentiles (16th centile) to approximate 1 (standard deviation) SD below the mean, adjusted for age and sex, derived from the composite memory or non-memory scores. The OCIND group included all who did not fulfil one or more diagnostic criteria for MCI. Since MCI was a small group, OCIND was regrouped with MCI in the final analysis.

*
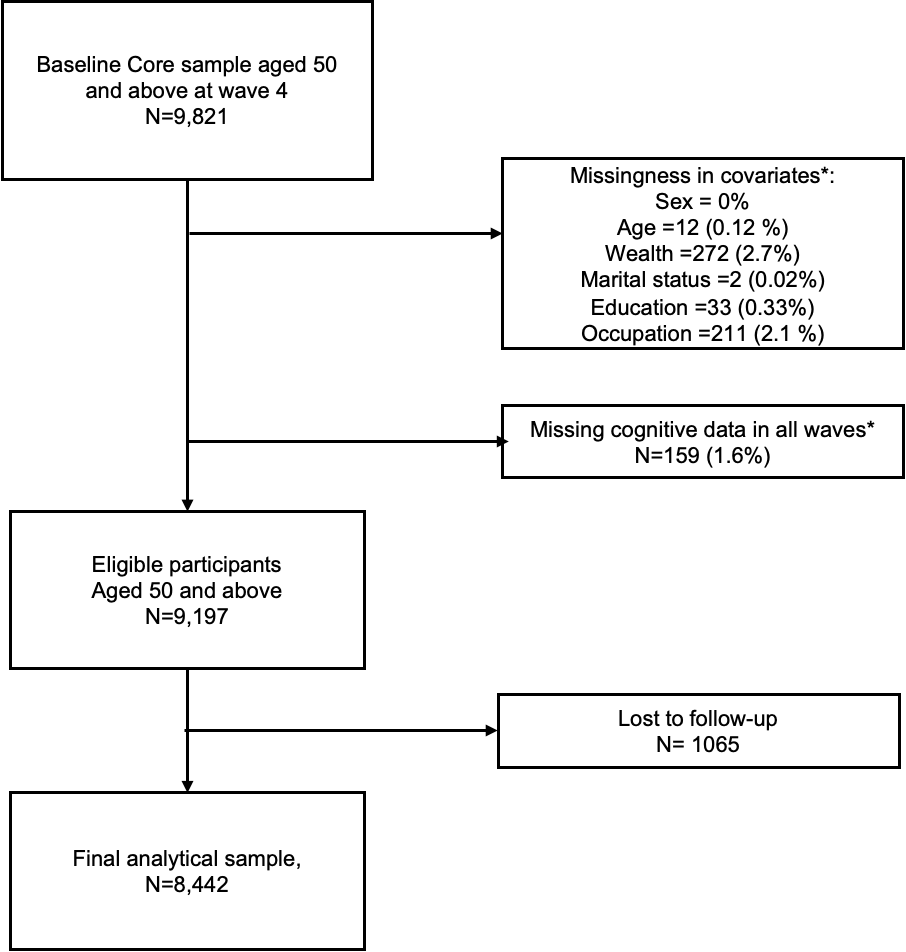
*

eFigureS2: Flowchart representing the sample selection

**Missingness is not mutually exclusive*

*Model fitness*

When we compared the predicted and observed prevalence of states over time, which is shown in Supplementary figure S2, a reasonably good match between the observed prevalence and the predicted prevalence was found.

**eFigure S3:** Observed vs. model predicted prevalence for each state over time, for unadjusted multistate Markov model

Supplementary Table S1: Transition probabilities and associated 95% confidence intervals (CI) for different education groups after 10 years

|  | NOCI to CIND | CIND to NOCI | NOCI to Dementia | CIND to Dementia | NOCI to Death | CIND to Death | Dementia to Death |  |
| --- | --- | --- | --- | --- | --- | --- | --- | --- |
| **Education** |  |  |  |  |  |  |  |  |
| Age 60 |  |  |  |  |  |  |  |  |
| Low | 0.42 (0.40,0.44) | 0.26 (0.24,0.28) | 0.04 (0.03,0.05) | 0.05 (0.04,0.07) | 0.17 (0.16,0.19) | 0.30(0.27,0.31) | 0.90 (0.81,0.96) |  |
| Middle | 0.36 (0.34,0.381 | 0.40 (0.38,0.425) | 0.02 (0.016,0.02) | 0.028 (0.02,0.03) | 0.19 (0.11,0.12) | 0.21 (0.20,0.23) | 0.86 (0.73,0.93) |  |
| High | 0.27 (0.25,0.29) | 0.51 (0.48,0.54) | 0.019 (0.013,0.027) | 0.027 (0.01,0.04) | 0.09 (0.08,0.10) | 0.20 (0.17,0.21) | 0.85 (0.70,0.95) |  |
|  |  |  |  |  |  |  |  |  |
| Age 80 |  |  |  |  |  |  |  |  |
| Low | 0.55 (0.53,0.57) | 0.090 (0.08,0.09) | 0.05 (0.04,0.06) | 0.05 (0.04,0.06) | 0.28 (0.27,0.30) | 0.35 (0.33,0.37) | 0.95(0.91,0.97) |  |
| Middle | 0.57 (0.55,0.58) | 0.16 (0.15,0.17) | 0.03(0.028,0.03) | 0.03 (0.02,0.03) | 0.20 (0.19,0.22) | 0.27 (0.26,0.30) | 0.92 (0.86,0.96) |  |
| High | 0.50 (0.47,0.52) | 0.24 (0.21,0.26) | 0.03 (0.02,0.04) | 0.03 (0.02,0.04) | 0.18 (0.16,0.20) | 0.26 (0.24,0.28) | 0.92 (0.81,0.97) |  |

*NOCI= No Cognitive Impairment, CIND= Cognitive Impairment, no dementia*

Supplementary Table S2: Transition probabilities and associated 95% confidence intervals (CI) for different occupation groups after 10 years

|  | NOCI to CIND | CIND to NOCI | NOCI to Dementia | CIND to Dementia | NOCI to Death | CIND to Death | Dementia to Death |
| --- | --- | --- | --- | --- | --- | --- | --- |
| **Occupation** |  |  |  |  |  |  |  |
| Age 60 |  |  |  |  |  |  |  |
| Routine/manual | 0.43 (0.40,0.44) | 0.31 (0.29,0.33) | 0.034 (0.02,0.04) | 0.043 (0.03,0.05) | 0.15 (0.14,0.16) | 0.26 (0.241,0.27) | 0.87 (0.760.94) |
| Intermediate | 0.43 (0.40,0.44) | 0.30 (0.28,0.33) | 0.03 (0.02,0.04) | 0.043 (0.03,0.05) | 0.15 (0.14,0.16) | 0.26(0.24,0.27) | 0.87 (0.77,0.94) |
| Managerial/  professional | 0.36 (0.34,0.37) | 0.39 (0.37,0.41) | 0.02 (0.02,0.03) | 0.032 (0.02,0.04) | 0.13 (0.13,0.14) | 0.23 (0.22,0.25) | 0.91 (0.80,0.97) |
|  |  |  |  |  |  |  |  |
| Age 80 |  |  |  |  |  |  |  |
| Routine/manual | 0.59 (0.57,0.60) | 0.098 (0.09,0.10) | 0.045 (0.03,0.05) | 0.04 (0.03,0.05) | 0.25 (0.23,0.27) | 0.31 (0.30,0.33) | 0.92 (0.87,0.95) |
| Intermediate | 0.59 (0.57,0.60) | 0.09 (0.10,0.10) | 0.04 (0.03,0.05) | 0.04 (0.03,0.05) | 0.25 (0.23,0.27) | 0.31 (0.29, 0.33) | 0.92(0.87,0.95) |
| Managerial/  professional | 0.56 (0.54,0.57) | 0.14 (0.12,0.15) | 0.03 (0.03,0.04) | 0.03 (0.03,0.04) | 0.24(0.22,0.25) | 0.30 (0.28,0.32) | 0.95 (0.90,0.97) |

*NOCI= No Cognitive Impairment, CIND= Cognitive Impairment, no dementia*

Supplementary Table S3: Transition probabilities and associated 95% confidence intervals (CI) for different wealth groups after 10 years

| **Wealth** | NOCI to CIND | CIND to NOCI | NOCI to Dementia | CIND to Dementia | NOCI to Death | CIND to Death | Dementia to Death |
| --- | --- | --- | --- | --- | --- | --- | --- |
| Age 60 |  |  |  |  |  |  |  |
| Low tertile | 0.41 (0.39,0.43) | 0.30 (0.28,0.32) | 0.03 (0.02,0.04) | 0.04 (0.03,0.05) | 0.15 (0.14,0.16) | 0.26 (0.25,0.28) | 0.89 (0.78,0.95) |
| Middle | 0.32 (0.30,0.34) | 0.43 (0.41,0.46) | 0.03 (0.02,0.04) | 0.023 (0.02,0.04) | 0.12 (0.11,0.13) | 0.21 (0.20,0.23) | 0.90(0.77,0.96) |
| Highest tertile | 0.26 (0.24,0.28) | 0.53 (0.51,0.56) | 0.01 (0.02,0.03) | 0.02 (0.01,0.03) | 0.09 (0.08,0.10) | 0.18 (0.17,0.20) | 0.87 (0.75,0.95) |
|  |  |  |  |  |  |  |  |
| Age 80 |  |  |  |  |  |  |  |
| Low tertile | 0.58 (0.56,0.5) | 0.09 (0.08,0.10) | 0.05 (0.04,0.06) | 0.05 (0.04,0.06) | 0.26 (0.24,0.27) | 0.32 (0.30,0.34) | 0.93 (0.89,0.96) |
| Middle | 0.55 (0.54,0.57) | 0.15 (0.14,0.17) | 0.03 (0.02,0.04) | 0.03 (0.02,0.04) | 0.22 (0.20,0.24) | 0.29 (0.27,0.31) | 0.93 (0.88,0.97) |
| Highest tertile | 0.52 (0.50,0.54) | 0.22 (0.20,0.24) | 0.03 (0.02,0.04) | 0.03 (0.02,0.04) | 0.18 (0.17,0.20) | 0.26 (0.24,0.27) | 0.92 (0.85,0.965) |

*NOCI= No Cognitive Impairment, CIND= Cognitive Impairment no dementia*

**Missing data**

Study members who had missing cognitive data were more likely to be older adults, had a lower educational level, had a manual/routine occupation or lowest levels of wealth· In terms of their gender, there were no significant differences in those providing cognitive data and those who did not (See Supplementary Table S4)

Supplementary Table S4: Difference in characteristics of the sample with complete and incomplete neurocognitive data

| Study variables | Mean (SD)/ % | |
| --- | --- | --- |
|  | Complete data | Missing data |
| ***Age*** | 67 (9.5) | 75.85 (11.76) |
|  |  |  |
| ***Sex*** |  |  |
| Male | 44.6 | 45 |
| Female | 55.4 | 55 |
|  |  |  |
| ***Marital status*** |  |  |
| Single/divorced | 34.5 | 37.9 |
| Married | 65.5 | 62.1 |
|  |  |  |
| ***Education*** |  |  |
| Low | 28.2 | 36.1 |
| Middle | 53.6 | 49.2 |
| High | 18.2 | 14.8 |
|  |  |  |
| ***Occupation*** |  |  |
| Routine and manual occupations | 31.4 | 36.1 |
| Intermediate occupations (non-manual) | 35.0 | 35.1 |
| Managerial and professional occupations | 33.6 | 28.9 |
|  |  |  |
| ***Wealth*** |  |  |
| Lowest Tertile | 33.4 | 39.8 |
| Middle | 33.6 | 33.4 |
| Highest Tertile | 33.0 | 26.8 |

Supplementary Table S5: Estimated standard errors (SE) of parameters from separate multistate models fitted to education, occupation, and wealth

| *Parameter* | *SE* | *Parameter* | *SE* | *Parameter* | *SE* |
| --- | --- | --- | --- | --- | --- |
| **Education** |  | **Occupation** |  | **Wealth** |  |
| NOCI to CIND |  | NOCI to CIND |  | NOCI to CIND |  |
| Intercept | 0.12 | Intercept | 0.118 | Intercept | 0.12 |
| Age (1-yr increment) | 0.00 | Age (1-yr increment) | 0.002 | Age (1-yr increment) | 0.00 |
| Sex (Male vs female) | 0.00 | Sex (Male vs female) | 0.002 | Sex (Male vs female) | 0.00 |
| Marital status (Not married vs married) | 0.00 | Marital status (Not married vs married) | 0.004 | Marital status (Not married vs married) | 0.00 |
| Education (middle level vs low level) | 0.03 | Wealth (Middle tertile vs lowest tertile) | 0.025 | Occupation (Intermediate vs routine/manual) | 0.02 |
| Education (High level vs low level) | 0.03 | Wealth (Highest tertile vs lowest tertile) | 0.028 | Occupation (Managerial/professional vs routine/manual) | 0.03 |
| **CIND to NOCI** |  | **CIND to NOCI** |  | **CIND to NOCI** |  |
| Intercept | 0.12 | Intercept | 0.117 | Intercept | 0.12 |
| Age (1-yr increment) | 0.03 | Age (1-yr increment) | 0.650 | Age (1-yr increment) | 0.65 |
| Sex (Male vs female) | 0.08 | Sex (Male vs female) | 0.035 | Sex (Male vs female) | 0.04 |
| Marital status (Not married vs married) | 0.03 | Marital status (Not married vs married) | 0.087 | Marital status (Not married vs married) | 0.09 |
| Education (middle level vs low level) | 0.72 | Wealth (Middle tertile vs lowest tertile) | 0.092 | Occupation (Intermediate vs Routine/manual) | 0.09 |
| Education (High level vs low level) | 0.03 | Wealth (Highest tertile vs lowest tertile) | 0.031 | Occupation (Managerial/professional vs Routine/manual) | 0.03 |
| **CIND to Dementia** |  | **CIND to Dementia** |  | **CIND to Dementia** |  |
| Intercept | 0.06 | Intercept | 0.068 | Intercept | 0.07 |
|  |  |  |  |  |  |
|  |  |  |  |  |  |
|  |  |  |  |  |  |
| Education (middle level vs low level) | 0.08 | Wealth (Middle tertile vs lowest tertile) | 0.622 | Occupation (Intermediate vs Routine/manual) | 0.03 |
| Education (High level vs· Low level) | 0.08 | Wealth (Highest tertile vs lowest tertile) | 0.035 | Occupation (Managerial/professional vs· Routine/manual) | 0.08 |
| **NOCI to Dementia** |  | **NOCI to Dementia** |  | **NOCI to Dementia** |  |
| Intercept | 0.63 | Intercept | 0.495 | Intercept | 0.48 |
| Education (Middle level vs low level) | 0.08 | Wealth (Middle tertile vs lowest tertile) | 0.080 | Occupation (Intermediate vs Routine/manual) | 0.08 |
| Education (High level vs low level) | 0.03 | Wealth (Highest tertile vs lowest tertile) | 0.031 | Occupation (Managerial/professional vs Routine/manual) | 0.03 |
| **Dementia to death** |  | **Dementia to death** |  | **Dementia to death** |  |
| Intercept | 0.36 | Intercept | 0.369 | Intercept | 0.37 |
| Age (1-yr increment) | 0.04 | Age (1-yr increment) | 0.097 | Age (1-yr increment) | 0.03 |
| Sex (Male vs female) | 0.80 | Sex (Male vs female) | 0.104 | Sex (Male vs female) | 0.61 |
| Marital status (Not married vs married) | 0.04 | Marital status (Not married vs married) | 0.027 | Marital status (Not married vs married) | 0.04 |
|  |  |  |  |  |  |
|  |  |  |  |  |  |

*Standard errors (SE),* *NOCI= No Cognitive Impairment, CIND= Cognitive Impairment no dementia*

| Supplementary Table S6: Sojourn Time and estimated standard errors (SE) of parameters with confidence intervals (CI) from separate multistate models fitted to education, occupation, and wealth at age 80 | | | | | |
| --- | --- | --- | --- | --- | --- |
| **EDUCATION** |  |  |  |  |  |
| Lowest Education level | | Sojourn time | SE | Lower CI | Upper CI |
| NOCI |  | 2.13 | 0.1 | 1.95 | 2.33 |
| CIND |  | 7.4 | 0.26 | 6.9 | 7.93 |
| Dementia |  | 3.36 | 0.33 | 2.77 | 4.07 |
|  |  |  |  |  |  |
| Middle |  |  |  |  |  |
| NOCI |  | 2.87 | 0.12 | 2.64 | 3.11 |
| CIND |  | 6.96 | 0.25 | 6.49 | 7.46 |
| Dementia |  | 3.95 | 0.46 | 3.14 | 4.96 |
|  |  |  |  |  |  |
| Highest Education level | |  |  |  |  |
| NOCI |  | 3.72 | 0.21 | 3.34 | 4.15 |
| CIND |  | 5.7 | 0.27 | 5.19 | 6.26 |
| Dementia |  | 3.98 | 0.8 | 2.68 | 5.91 |
| **OCCUPATION** |  |  |  |  |  |
| Routine/manual |  |  |  |  |  |
| NOCI |  | 2.2 | 0.1 | 2.01 | 2.4 |
| CIND |  | 7.9 | 0.28 | 7.37 | 8.47 |
| Dementia |  | 3.93 | 0.44 | 3.16 | 4.88 |
| Intermediate |  |  |  |  |  |
| NOCI |  | 2.56 | 0.11 | 2.35 | 2.79 |
| CIND |  | 6.74 | 0.25 | 6.27 | 7.25 |
| Dementia |  | 3.32 | 0.4 | 2.62 | 4.2 |
| Managerial/professional |  |  |  |  |  |
| NOCI |  | 3.21 | 0.15 | 2.93 | 3.51 |
| CIND |  | 6.1 | 0.24 | 5.64 | 6.6 |
| Dementia |  | 4 | 0.56 | 3.05 | 5.26 |
| **WEALTH** |  |  |  |  |  |
| Lowest wealth tertile | |  |  |  |  |
| NOCI |  | 2.21 | 0.1 | 2.03 | 2.41 |
| CIND |  | 8.04 | 0.28 | 7.52 | 8.6 |
| Dementia |  | 3.67 | 0.38 | 2.99 | 4.51 |
| Middle wealth tertile | |  |  |  |  |
| NOCI |  | 2.7 | 0.12 | 2.47 | 2.94 |
| CIND |  | 6.53 | 0.25 | 6.06 | 7.05 |
| Dementia |  | 3.56 | 0.46 | 2.75 | 4.59 |
| Highest wealth tertile | |  |  |  |  |
| NOCI |  | 3.3 | 0.16 | 3.01 | 3.62 |
| CIND |  | 5.75 | 0.24 | 5.29 | 6.24 |
| Dementia |  | 3.84 | 0.57 | 2.87 | 5.14 |

***References***

1. Abner EL, Kryscio RJ, Cooper GE, et al. Mild cognitive impairment: statistical models of transition using longitudinal clinical data. *Int J Alzheimers Dis* 2012; **2012**: 291920-.

2. Hout Avd. Multi-state survival models for interval-censored data / Ardo van den Hout. Boca Raton: CRC Press; 2017.

3. Meira-Machado L, de Uña-Alvarez J, Cadarso-Suárez C, Andersen PK. Multi-state models for the analysis of time-to-event data. *Statistical methods in medical research* 2009; **18**(2): 195-222.

4. Titman AC. Flexible Nonhomogeneous Markov Models for Panel Observed Data. *Biometrics* 2011; **67**(3): 780-7.
